# Comparing yield and relative costs of WHO TB screening algorithms in selected risk groups among people aged 65 years and over in China, 2013

**DOI:** 10.1371/journal.pone.0176581

**Published:** 2017-06-08

**Authors:** Canyou Zhang, Yunzhou Ruan, Jun Cheng, Fei Zhao, Yinyin Xia, Hui Zhang, Ewan Wilkinson, Mrinalini Das, Jie Li, Wei Chen, Dongmei Hu, Kathiresan Jeyashree, Lixia Wang

**Affiliations:** 1 National Center for Tuberculosis Control and Prevention, Chinese Center for Disease Control and Prevention, Beijing, China; 2 Institute of Medicine, University of Chester, Chester, United Kingdom; 3 Médecins Sans Frontières (MSF), Delhi, India; 4 Velammal Medical College Hospital & Research Institute, Madurai, India; Chinese Academy of Medical Sciences and Peking Union Medical College, CHINA

## Abstract

**Objective:**

To calculate the yield and cost per diagnosed tuberculosis (TB) case for three World Health Organization screening algorithms and one using the Chinese National TB program (NTP) TB suspect definitions, using data from a TB prevalence survey of people aged 65 years and over in China, 2013.

**Methods:**

This was an analytic study using data from the above survey. Risk groups were defined and the prevalence of new TB cases in each group calculated. Costs of each screening component were used to give indicative costs per case detected. Yield, number needed to screen (NNS) and cost per case were used to assess the algorithms.

**Findings:**

The prevalence survey identified 172 new TB cases in 34,250 participants. Prevalence varied greatly in different groups, from 131/100,000 to 4651/ 100,000. Two groups were chosen to compare the algorithms. The medium-risk group (living in a rural area: men, or previous TB case, or close contact or a BMI <18.5, or tobacco user) had appreciably higher cost per case (USD 221, 298 and 963) in the three algorithms than the high-risk group (all previous TB cases, all close contacts). (USD 72, 108 and 309) but detected two to four times more TB cases in the population. Using a Chest x-ray as the initial screening tool in the medium risk group cost the most (USD 963), and detected 67% of all the new cases. Using the NTP definition of TB suspects made little difference.

**Conclusions:**

To “End TB”, many more TB cases have to be identified. Screening only the highest risk groups identified under 14% of the undetected cases,. To “End TB”, medium risk groups will need to be screened. Using a CXR for initial screening results in a much higher yield, at what should be an acceptable cost.

## Introduction

Tuberculosis (TB) is still a major global health problem and has been identified in the Sustainable Development Goals as one of the major diseases to be eliminated by 2030. Recent estimates from World Health Organization (WHO) give the global prevalence of TB as 174/100,000 and the incidence as 133/100,000 [1].

Despite China having a much lower prevalence (89/100,000) and incidence (68/100,000), there were still 1,200,000 TB cases and 930,000 incident TB cases in 2015. This accounted for almost 10% of the estimated new cases worldwide [1]. China therefore has one of the highest burdens of TB globally.

Passive case finding (PCF) and treatment of diagnosed TB disease are currently the principal means globally and in China, of controlling transmission of *Mycobacterium tuberculosis* and reducing TB incidence [2,3]. The standard PCF approach has not been successful in detecting all cases and globally it has been estimated that nearly 37% of new TB cases are undiagnosed or not reported [1].

Active case finding (ACF) is believed to contribute to the earlier detection of persons with TB and an earlier initiation of treatment, and to result in better outcomes for individuals with reduced transmission in the community [4–6]. Almost all ACF interventions rely on sputum smear-microscopy as the basis for diagnosis; but there is also growing evidence that screening through the use of chest radiographs is both effective and cost-effective in high-burden settings [7,8].

The results of most ACF studies show a predictable rise in the number of TB cases identified [9,10]. However, the individual and community-level benefits from active screening for TB disease remain uncertain, and the benefits of earlier diagnosis on patient outcomes and on-going TB transmission have not yet been established [11].

WHO recently published operational guidelines on systematic screening for active tuberculosis [12,13]. These have been developed for use in settings with different diagnostic resources, and they acknowledge that the yield (number of new cases of PTB found by each screening algorithm) will vary depending on the prevalence of undiagnosed TB and will be greater in subgroups at higher risk of TB. The prioritization of high risk groups for screening should be based on potential benefits and harms, the feasibility of the initiative, the acceptability of the approach, the number needed to screen, and the cost of screening[12,13].

In China, the population of persons aged 65 years and over is rapidly expanding. They are at increased risk of TB due to a longer exposure to infection with *Mycobacterium tuberculosis* and declining immunity with age which allows latent infection to reactivate and cause disease[14,15]. Those 60 years and over have a high prevalence of TB (349/100,000). This is 2.6 times higher than those aged 45 to 59 [16]. Symptoms of TB may be non-specific or absent, and attendance at health facilities may be erratic [17].

The aim of our study was to compare four screening algorithms for TB in persons aged 65 years and over. Using data from a TB prevalence study conducted in China, we sought to identify the risk groups with the highest yield and determine the relative costs of the different algorithms.

## Methods

### Ethical considerations

The prevalence study was reviewed and approved by the Institutional Review Board of Chinese Center for Disease Control and Prevention before commencing data collection. This study using data collected in the prevalence study, was approved by the Ethics Advisory Group of the International Union against Tuberculosis and Lung Disease, Paris, France.

### Study design

This was an analytic study based on data from a cross-sectional study.

### Study setting

China has a population of 1.37 billion, of whom 10.1% are people aged 65 years or over, and a GDP per capita of $7590 [18]. The level of affluence and urbanization varies greatly across the country.

There is a National TB control program (NTP) which develops the national protocols for detection and treatment of TB. The diagnosis of TB, including microscopy and X-ray examination, first-line anti-TB drugs and DOTS are all offered free of cost.

### Study population

All persons aged 65 years and over who were interviewed in the TB prevalence study were included in the study.

### TB prevalence survey

A TB prevalence survey in adults was conducted in 2013, the results of which will be published in full elsewhere. Sample size was estimated using a method appropriate to estimate a single population proportion. The 369/100,000 prevalence of bacteria-positive PTB among elderly people (≥65 years) from the latest national tuberculosis prevalence survey was used as a reference. 95% confidence level and 0.2 allowable error were assumed. The formula was *n* = *pq*/(*d*/z_α_)^2^ (p = 369/100,000, q = 1-p, d = 0.2p,α = 0.05, *Z*_α_ = 1.96,). A total of 25,931 elderly participants were requested. In consideration of 10% non-response, sample size should be 28,812.

**Fig 1:**
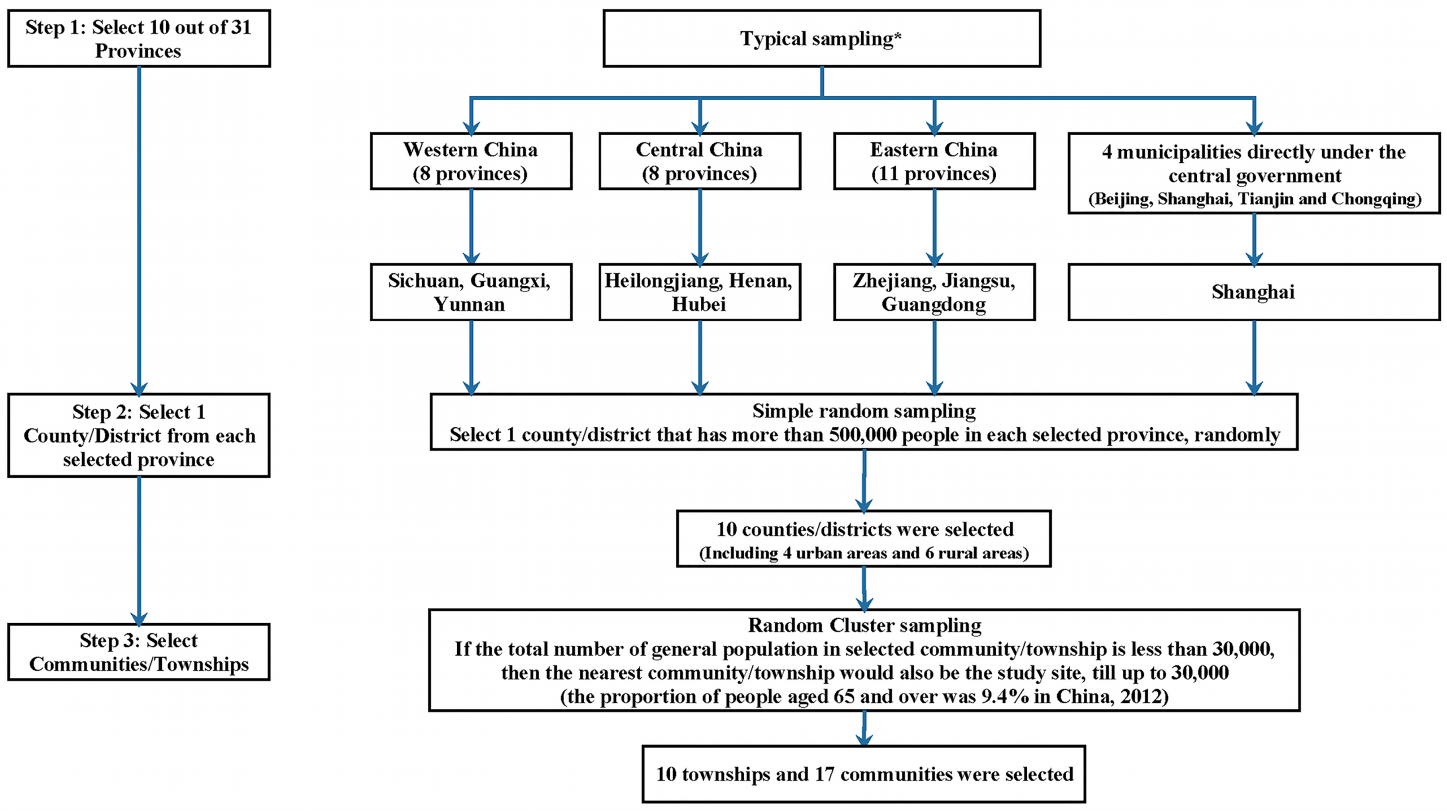
The sampling procedure of the TB prevalence survey in China in 2013. (*) 10 out of 31 provinces were selected, by considering the cooperative willingness, human resources and related abilities of each province.

**Fig 2:**
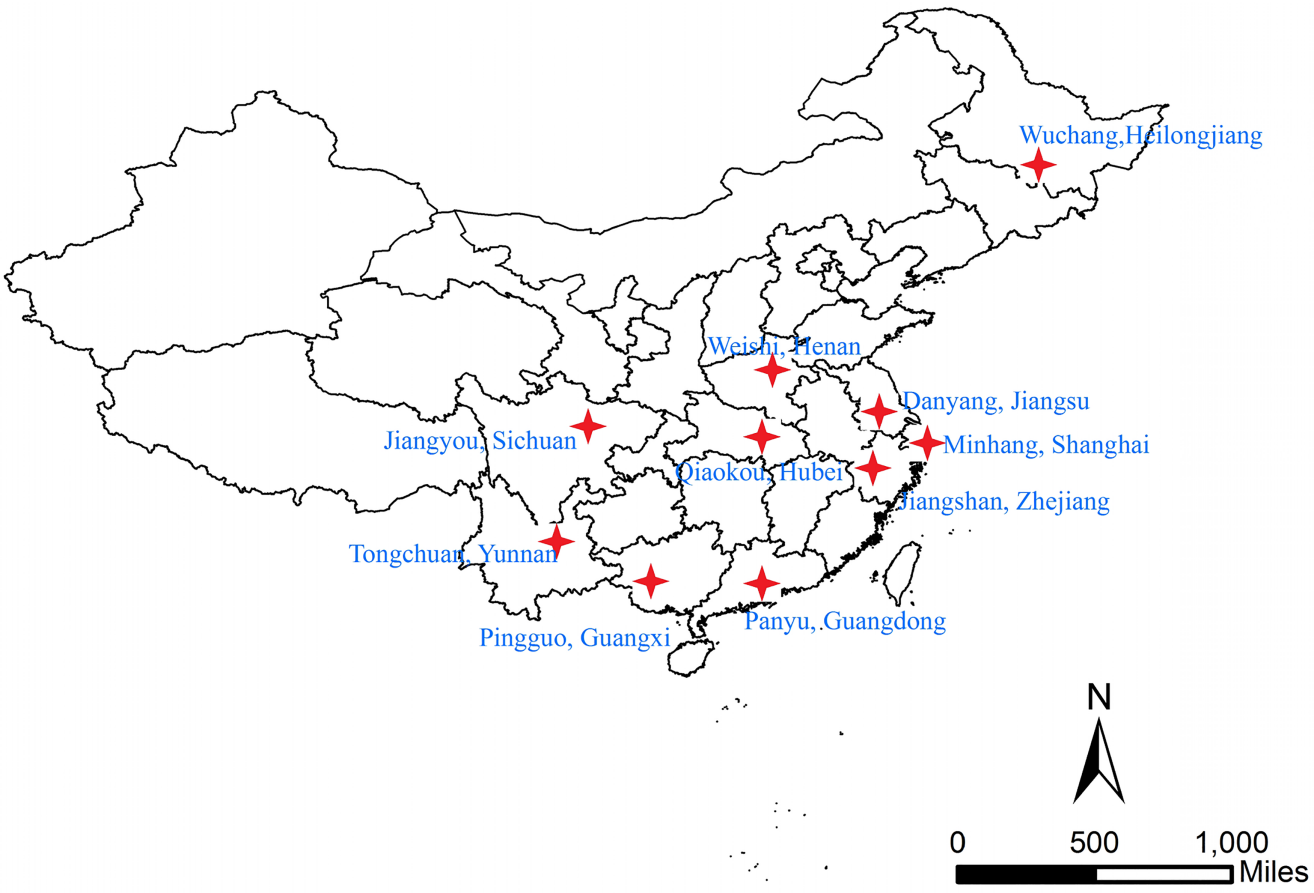
The location of 10 sample counties in the TB prevalence survey in China in 2013.

Multi-stage cluster sampling was used, and the procedure of sampling is shown in Fig 1. 27 study fields (10 townships and 17 communities) from 10 counties of 10 provinces were selected. The 10 counties are shown in Fig 2. The chosen number of townships or communities of each county depended on the population of aged people. Finally, 38,888 participants were included in the study.

Within this survey, each resident aged over 65 years was interviewed by trained staff face-to-face in their home using a standard questionnaire. Data on participants’ sex, age, marital status, education, past medical history, occupation, tobacco and alcohol use were collected. Their height and weight were used to calculate their Body Mass Index (BMI).

All participants were invited to have a full-size chest X-ray (CXR). Participants who had any suspected TB symptoms (cough for over 2 weeks or haemopytsis) or abnormal lung field shadows on CXR, were requested to submit 3 sputum samples (morning, night and spot sputum). The sputum samples were submitted and examined by smear microscopy for acid-fast bacilli and were assessed for mycobacterial culture using the solid Löwenstein—Jensen medium.

The cost of each component of the screening process is shown in Table 1. The cost of the household visits was based on the additional daily allowance, agreed nationally for work relating to infectious diseases, divided by the average number of households visited in a day. The price of a CXR, sputum smear and culture was the average market price paid.

**Table 1:** Cost of each component of active TB case-finding in this study in China in 2013.

Contents	Cost per unit (USD)
Household primary screening by village health workers	0.15
Chest X-ray	9.0
Sputum smear	3.9
Sputum culture	4.8

Pulmonary TB was diagnosed as sputum smear-positive and/or culture positive, or it was diagnosed on CXR based on a decision by a group of clinical doctors and radiologists [16]. Quality checks were done according to the National Guidelines [19].

The data collected in the survey were double entered using an online input system developed by a local software company.

### TB screening algorithms

WHO has published three different algorithms for use depending on the risk groups and the diagnostic resources available [12]. These WHO algorithms (A1, A1b, A2 and A3) are shown in Table 2. Algorithm A1b is similar to A1 but is altered to reflect national policy in China which is to screen those with cough for more than 2 weeks and/or haemoptysis, rather than cough alone.

**Table 2:** Algorithms to screen the population for TB aged 65 or over in the different high risk group in this study, based on the WHO recommendations.

Algorithms	Intervention 1	Intervention 2	Intervention 3	Intervention 4
WHO A1	Interview	CXR	Smear	Culture
	If cough lasting > 2 weeks, then	If positive, then	If positive = TB	If positive = TB
			If negative, then	If negative, then possible clinical diagnosis with CXR
WHO A1b	Interview	CXR	Smear	Culture
	If cough lasting > 2 weeks &/or haemoptysis, then	If positive, then	If positive = TB	If positive = TB
			If negative, then	If negative, then possible clinical diagnosis with CXR
WHO A2	Interview	CXR	Smear	Culture
	If any TB symptoms(cough of any duration, haemoptysis, weight loss, fever, night sweats), then	If positive, then	If positive = TB	If positive = TB
			If negative, then	If negative, then possible clinical diagnosis with CXR
WHO A3	CXR	Smear	Culture	NA
	If positive, then	If positive = TB	If positive = TB	
		If negative, then	If negative, then possible clinical diagnosis with CXR	

The high risk groups [12] for TB identified from the literature are shown in Table 3.

**Table 3:** Definition of high risk factors for TB used in this study.

**Previous TB cases**: registered in TB Management Information System, and finished treatment or cured.
**HIV/AIDS**: registered in local CDC database.
**Known Diabetes**: recorded on the Citizen Health Management Files as diagnosed with Diabetes, plus those using medicine to control Blood glucose by self-report.
**Close Contacts**: living with new active PTB case for at least 7 days in the three months before diagnosis.
**BMI<18.5**: Weight (kg)/Height2 (m2) <18.5.
**Tobacco use**: ever smoked tobacco by self-report.
**Drinking history**: drinking more than one unit (21 grams pure alcohol) per week.

### Data analysis

We obtained the point prevalence (number of missing TB cases detected/ population screened) data from the TB prevalence study and transferred this into our electronic database. For each algorithm and for different high risk groups, we calculated the yield of TB screening.

Yield = number new TB cases (smear-positive PTB, culture-positive PTB and active TB)

Number needed to screen to detect one case (NNS) = total number screened / number of cases identified

The costs of each of the algorithms were applied to the number of TB cases diagnosed to give indicative costs per case of TB detected: this was done by dividing the relative total cost of the tests in the algorithm by the number of new cases of TB identified.

All tests were performed using SAS 9.3 (SAS Institute Inc., USA).

## Results

From the TB prevalence survey, there were 38,888 eligible people aged 65 years and over in the ten sample areas. Demographic characteristics are shown in Table 4.

**Table 4:** Demographic characteristics of the population aged 65 or over in the sample population in China in 2013.

Characteristics	No.	%
**Total**	38,888	100.0
**Sex**		
Male	18,005	46.3
Female	20,883	53.7
**Age group**		
65–74	24,102	62.0
75–84	12,193	31.3
85-	2,593	6.7
**Place of residence**		
Urban	13,533	34.8
Rural	25,355	65.2

Nineteen people were excluded as they were known TB cases under treatment. 4,619 refused to participate. 34,250 (88.1%) agreed to participate in this study. Of those who agreed to participate 33,510 (97.8%) had a chest X-ray, and 1,534 submitted sputum for smear and culture.

The number of people diagnosed with TB by smear and/or culture is shown in Table 5. There were 172 new TB cases, of which 116 were diagnosed only by CXR and clinical diagnosis.

**Table 5:** Total number of new TB cases found in the study population in China in 2013 and how diagnosed, smear positive TB and/or culture positive TB, or CXR and clinical alone.

		culture		Total
		+	-	
Smear	+	23	8	31
	-	25	116	141
Total		48	124	172

The number, yield, and prevalence of new TB cases, in each risk group is shown in Table 6. The prevalence of new TB cases in males was 3 times higher than in females. The prevalence rates in “previous TB” and “close contacts” were very high, 3,698 and 3,192/100,000 respectively. Also, the groups “BMI<18.5” and “Tobacco use” had high prevalence of new TB cases. For all risk groups except “BMI<18.5”, the new TB case prevalence in “rural areas” was 2 to 3 times higher than that in “urban areas”

**Table 6:** Number in each risk group, number of new TB cases diagnosed and, prevalence of new TB cases, in the prevalence survey China, 2013.

Groups	Total			Urban areas			Rural areas		
	No. in risk group	No. of TB diagnosed	Prevalence of new TB cases (1/100,000)	No. in risk group	No. of TB diagnosed	Prevalence of new TB cases (1/100,000)	No. in risk group	No. of TB diagnosed	Prevalence of new TB cases (1/100,000)
All aged 65 and over	34250	172	502	12932	34	263	21318	138	647
Previous TB	595	22	3698	251	6	2390	344	16	4651
Close contacts	94	3	3192	20	0	0	74	3	4054
BMI<18.5	3632	39	1074	931	9	967	2701	30	1111
Tobacco use	6763	55	813	2168	12	554	4595	43	936
Male	16044	129	804	6044	25	414	10000	104	1040
Alcohol use	6543	40	611	1907	6	315	4636	34	733
Diabetes	2400	14	583	1306	3	230	1094	11	1006
Female	18206	43	236	6888	9	131	11318	34	300
HIV/AIDS	1	0	0	0	0	—	1	0	0

Two specimen groups at increased risk of TB were identified to run the WHO algorithms. Group 1 “medium risk” was a group of 12,006, with a prevalence (between 936/100,000 and 4,651/100,000). This medium risk group comprised those living in a “rural area”, who were “men” or a “previous TB case”, or were a “close TB contact” or a “BMI <18.5” or “tobacco users”.

Group 2 “high risk” totalled 668 people, comprised the groups with the highest prevalence (over 3,000/100,000), which were all “previous TB cases” and all “close TB contacts”. (Table 7)

**Table 7:** Risk group 1 and 2, and yield, and prevalence of new TB cases, for each group.

Groups	No. in risk group	No. of new TB cases diagnosed	Prevalence of new TB cases (per 100,000)
medium risk group 1*	12006	119	991
high risk group 2**	688	25	3,634

*Group 1 medium risk: Living in a rural area and male, or previous TB, or close contacts, or BMI<18.5, or tobacco use

**Group 2 high risk: previous TB or close contacts.

The numbers of each tests used in the screening algorithm, number of new TB diagnosed and relative cost per case for each group are shown in Table 8.

**Table 8:** Number of tests to be taken, yield and cost per case for each algorithm of the medium risk group 1, high risk group 2 and all aged 65 and over.

Algorithms	Group 1*						Group 2**						All aged 65 and over					
	No. of CXR	No. of Smear	No. of Culture	No. of new TB diagnosed	NNS	Cost per case (USD)	No. of CXR	No. of Smear	No. of Culture	No. of new TB diagnosed	NNS	Cost per case (USD)	No. of CXR	No. of Smear	No. of Culture	No. of new TB diagnosed	NNS	Cost per case (USD)
WHO A1	366	74	62	25	481	221	59	31	25	12	58	72	611	103	90	31	1,105	330
WHO A1b	386	79	67	26	462	221	61	32	26	12	58	74	643	110	97	32	1,071	331
WHO A2	683	107	94	29	414	298	97	41	35	12	58	108	1313	153	138	37	926	458
WHO A3	11953	551	531	116	104	963	677	160	154	24	29	309	33510	989	959	164	209	1,881

*Group 1 medium risk: Living in a rural area and male, or previous TB, or close contacts, or BMI<18.5, or tobacco use.

**Group 2 high risk: previous TB or close contacts.

The yield for algorithms WHO 1, 1b and 2 increased slightly from 15% (25/172) to 17% (29/172) in the medium risk group with cost per case increasing from $221 to $298 and was unchanged in the high risk group, 7% (12/172) at a cost of between $72 and $108 per case. For all aged 65 and over, algorithms WHO 1, 1b and 2 found 18% (31/172) to 22% (37/172) at a cost of between $330 and $458 per case.

WHO A3 diagnosed 67% (116/172), 14% (24/172) and 95% (164/172) of new TB cases respectively in two risk groups and all aged 65 and over, which were two and five times as many new TB cases as the other three algorithms, but the cost per case was three to five times higher at $963, $309 and $1,881 per case respectively.

## Discussion

This is the first study that has applied the WHO TB algorithms to population data of a country. The study discussed the yield and relative cost per case when each algorithm was applied on a population aged 65 years and over.

The prevalence survey demonstrates the difficulty of screening 100% of a population. Only 88% agreed to participate and of those a small proportion did not attend to have a CXR.

Using the WHO algorithms, the NNS and the cost per case detected, varies depending on the prevalence in risk groups and which algorithm is used. But the lower cost per case detected may leave up to 93% of new cases undetected.

Using CXR as the first screening test as in algorithm WHO A3 in the medium risk group detects a much higher proportion of the new TB cases in the whole population (116/ 172) than the other algorithms (25 to 29/172) at a cost of $963 per case.

The strengths of this study were that it used a large dataset collected as part of a carefully designed and implemented survey, which used the current TB diagnostic protocols and tests in China for diagnosis. Using real data to model the WHO algorithms showed how they work in practice. The study followed the Strengthening the Reporting of Observational Studies in Epidemiology (STROBE) guidelines [20] and sound ethics principles for the conduct and reporting of this study [21].

The study had a few limitations. The costing data that was collected was basic and was only indicative of the relative costs of the different groups being screened. Only current occupation was recorded and most participants were retired, thus risk groups based on previous occupation could not be identified. The original prevalence survey identified 172 new cases of TB and this has been used in this study. This is likely be incorrect for several reasons 1) the reported sensitivity of CXR as a screening tool is 87% and the specificity 89% [12]. 2) There will also be false positives included particularly as 67% of the new diagnoses were not confirmed by smear or culture. 3) Only 88% of the target population was screened. In our study, no modeling of screening of people living with HIV was possible, as in the data there was only one person who was living with HIV.

Screening the high risk group only, of “previous TB cases” and “close contacts”, gives a low cost per case. Studies from Africa and Cambodia also confirmed it was cost-effective to implement ACF among close contacts but the proportion of undiagnosed cases detected in the population is only 7% [22,23]. If the End TB target is to be achieved this is not effective.

It is being argued that the Stop TB proposal of ACF costing $350 per case [24] is too low to enable enough TB cases to be identified to reduce the prevalence in the community. It has been suggested that $1000 per case is more realistic [24] and is more similar to the cost and benefit of ART.

Use of WHO algorithm 3 in China would detect 67% of the new cases in the elderly community, at a crude cost of under $1000. The actual cost will be higher, but if the screening was set up as a large scale program the cost per test may well markedly reduce, for example with increased use of digital CXR [25].

The National Project of Basic Public Health Service launched by Ministry of Health in 2011 has made it easier for China to implement ACF [26]. In this project, all elderly people have an annual interview and physical examination, and the information recorded in the citizen health management file. This means that high risk groups as in Table 2 can be identified from routine data, and ACF can be combined with the annual physical examination. A pilot study in China, which integrated TB screening into annual health examinations for the rural elderly, and targeted diabetes patients and close contacts, had a significant yield. But no TB case was identified from close contacts alone [27].

China NTP used a different definition of TB symptoms from those in the WHO algorithm in that it uses “cough for 2 weeks or more, and haemoptysis”. This was used in the algorithm WHO A1b. The study results found there was little difference in the number of cases detected from using cough alone. Three more cases were identified when weight loss, fever and night sweats were added in algorithm 2. This shows that it is not necessary to change the nationally agreed TB symptoms used for screening.

Algorithm WHO A1 and WHO A2, using symptoms as the initial screening, will miss many undiagnosed TB cases when implemented in China, but it can still be used in some resource-limited areas, such as Western China.

This study has implications for other TB high burden countries which are also resource-limited, such as India and Indonesia. Choosing the optimal ACF strategy depends on the TB prevalence, economics, and human resources, etc. and it needs to fit with local health policies and available technology. This study has shown how the yield varies greatly and higher costs may need to be accepted in order to have an impact on the burden of TB.

To achieve the ambitious targets of ending the TB epidemic by 2035, ACF screening has to be implemented more widely.

## Conclusions

WHO recommends that indiscriminate mass screening should be avoided, and the prioritization of risk groups for screening should be based on the prevalence of new cases [12]. Knowing the expected prevalence of TB in risk groups enables appropriate targeting of screening and in China risk groups can be identified from routine data. The cost per diagnosed case, and NNS increases as the prevalence reduces. However if just the highest risk groups are screened, only between 7 and 14% of the undetected cases will be found, depending on the algorithm used. To “End TB”, appropriate medium risk groups will need to be screened. To obtain the highest yield, a CXR should be used for initial screening, as in WHO Algorithm 3.
